# Controversies Regarding Neonatal Inguinal Hernia

**Published:** 2014-07-10

**Authors:** V Raveenthiran

**Affiliations:** Department of Pediatric Surgery, SRM Medical College and Hospital SRM University, Chennai, India.

**Introduction**

Athena used to think that neonates are altogether a different species than Homo sapiens. For example, they are poikilothermic in contrast to homeothermic adult human beings. Inguinal hernia is yet another proof of this philosophical assertion. Herniotomy, a minor affair in grown-up children, is often a serious issue in newborn especially when they are premature. Neonatal inguinal hernia is also peculiar that controversies regarding it refuse to die down despite the pertinent facts being well established. Athena reviewed the literature to know the recent status of these controversies. 

**Role of Laparoscopy**

The most sensational of all controversies is the role of laparoscopy in neonatal hernia repair. Even in older children, opinion is divided regarding the usefulness of laparoscopic herniotomy. Laparoscopy converts what is essentially a subcutaneous procedure into invasive intra-abdominal operation. Open herniotomy can be done under spinal or caudal analgesia while laparoscopy mandatorily requires general anesthesia thereby increases the risk of post-operative apnea. Scar of open surgery is invisible even in swimsuit while laparoscopy leaves unsightly scars in mid-abdomen. Open herniotomy is a day-care procedure thereby annuls the “early discharge” benefits of laparoscopy. On the other hand, investment cost of instruments, prolonged operating time and significant learning curve are disadvantages of laparoscopy. Therefore, a group of pediatric surgeons denounce laparoscopic herniotomy in any age group. Conversely, laparoscopic enthusiasts claim that it facilitates contralateral exploration thereby eliminates the need of a second operation should contralateral recurrence occurs. Optical magnification is also said to reduce the risk of operative injuries to vas deference. Additional concerns of laparoscopy in newborn are adversities of pneumoperitoneum, impaired gas exchange in immature lungs, fragility of peritoneal sac and limited work-space for intra-abdominal maneuvering. 

Unfortunately, there are no well-designed, statistically robust, controlled studies to settle the controversies. Only half-a-dozen publications [1-5] describe subjective experience with neonatal laparoscopic herniotomy. Pastore and Bartoli [1] studied 30 neonates, 11 of whom had bilateral inguinal hernia. 93% of their patients were premature. The mean operating time was 30 min (range 20 - 35 min) for unilateral hernia and 41 min (range 26 - 51 min) for bilateral hernia. There were no intra- or post-operative complications. At a mean follow-up of 21 months (range 6 - 40 months) there were no recurrences. Chan et al [2] reported 79 premature newborn that underwent laparoscopic hernia repair. Nearly 65% of them had bilateral hernia. The mean operating time was 46 min (range 17 - 93 min). Immediate results were gratifying without any major complications. Nevertheless, 1.3% recurrence at a mean follow-up 26 months could be a cause of concern. As the maximum follow-up was only 84 months, it would be too early to assess and comment on post-herniotomy atrophy of the testis. 

The other 3 papers [3-5] are from the department of Felix Schier and hence Athena could sense “salami slicing” and overlap of data between them. She read and interpreted them in cohesion. Anesthesia related complications were noted in 5 to 12 %. At a median follow-up of 26 months, the risk of recurrent hernia is increased by 14%. [4] Although no testicular atrophy was registered at 6 - 52 months of follow-up, about 6% of the infants required subsequent orchidopexy. [4] Purse-string closure of internal ring appears to cause holding-up of the testis by entangling its vaso-vasal pedicle. It is not very clear if the high-lying testis is the result of arrested normal-descend or the result of testicular ascend following purse-string closure of ring. Risk of high-lying (? undescended) testis is increased by 67% for every 1 kg less weight of the newborn. [5] Athena, in her wisdom, would avoid laparoscopic herniotomy in newborn until scientifically more robust data emerge in support of it.

**Optimal Timing of Surgical Repair**

Optimal timing of surgical repair is yet another controversy especially in premature neonates. As much as 63% of American pediatric surgeons advocate early surgical repair citing the risk of strangulation and testicular atrophy during the waiting period. [6] They typically would repair the hernia before the neonate is discharged from nursery. [7] Another school of pediatric surgeons would electively schedule the operation contending better tolerance of anesthesia with postnatal maturation. [8] They typically would wait until the infant reaches 2.5 to 3 kg weight before embarking upon surgical repair. They justify the policy of waiting, as the risk of incarceration is lesser in premature neonates (13 - 18%) than that of full term neonates (28 - 31%). [9] 

Analyzing 1123 herniotomies in premature neonates, Lautz et al [10] found that the risk of strangulation was 9% at the post-conceptional age (PCA) of 36 - 39 weeks while it increases more than two-fold (21%) at PCA > 40 weeks. Risk of incarceration was 16% when the hernia is repaired during birth hospitalization, while it increased to 28% if the operation is done during a subsequent admission within one year. Vaos et al [9] took the argument further by advocating surgical repair within one week of diagnosis. Among 41 premature neonates, they compared 25 herniotomies that had been done within one week of diagnosis (the short waiting group - SWG) with 16 repairs that had been done after one week (the long waiting group - LWG). Rate of incarceration was significantly higher in LWG (56%) than that of SWG (12%). Recurrence rate was 5% in SWG as compared to 31% in LWG. Testicular atrophy was 5% in SWG, while it was as high as 23% in LWG. There was also one death in LWG, although it was not statistically significant. Interestingly, the mean operating time was significantly less in SWG (35+5 min) than LWG (47+11 min). This difference is attributed to easy dissection of vas soon after birth. High levels of maternal progesterone appear to facilitate easy separation of vas from fragile hernial sac. 

Contrary to the foregoing descriptions, Takahashi et al [11] compared 14 herniotomies done before discharge from nursery and 33 operations done during subsequent admission. They noted incarceration was less frequent and delayed extubation was more frequent in preterm infants as compared to term neonates. Lee et al [12] studied 45 hernia repairs done before discharge from neonatal intensive care unit (NICU) and 35 repairs done electively after discharge. None of the infants of “delayed repair” group had incarceration while 11% of those operated early had strangulation. Risk of apnea was similar in both the groups. The median post-operative hospital stay for “early repair” group was 8 days, while it was only one day for “delayed repair” group. Lee et al [12] concluded that delayed elective repair is superior to early repair. Athena wonders if the authors have reversed cause-effect relationship while interpreting the data. For example, increased incidence of incarceration in “early repair” group could be the cause as to why they are in that group. 

Despite the ongoing controversies, Athena would repair inguinal hernia in newborn (preterm or otherwise) as soon as the infant is medically fit enough to withstand anesthesia. 

**Overnight admission for apnea monitoring**

Traditionally, the incidence of apnea in preterm neonates undergoing herniotomy is cited as 49%. [12, 13] Overnight admission and apnea monitoring is recommended in this cohort. However, recent studies suggest that the risk of apnea has reduced to 5% owing to advances in anesthesiology. [12] Therefore, a subset of pediatric surgeons would not insist upon overnight hospitalization. While pondering this issue, nature of anesthesia and post-operative analgesia should be considered as co-variables. Narcotic analgesia, general anesthesia and prolonged operating time are well known to be associated with post-operative apnea.

Laituri et al [13] divided 363 preterm herniotomies into 3 groups according to PCA as less than 40 weeks, 40-49 weeks and 50-60 weeks. The lesser the PCA the more frequent was apnea (44% vs 21% vs 16% respectively) and oxygen desaturation (57% vs 43% vs 30% respectively). Therefore, they recommended overnight hospital stay for preterm infants less than 50 weeks of PCA. 

Ozdemir et al [14] reached a similar conclusion by studying 428 formerly preterm infants. Apnea was noted in 4.7% of infants less than 45 weeks of PCA while it was only 0.8% at PCA of 45 - 60 weeks. In older infants, co-morbidities such as anemia, necrotizing enterocolitis and bronchopulmonry dysplasia predispose to apnea. They recommended overnight observation in all infants of PCA less than 45 weeks while being selective in others.

Murphy et al [15] recorded apnea in only 6 of the 126 (4.8%) premature infants undergoing herniotomy. Low birth weight, low gestational age, previous episodes of apnea, presence of co-morbidities (such as intraventircular hemorrhage and patent ductus arteriosus - but not bronchopulmonary dysplasia) and need for mechanical ventilation or oxygen supplementation at birth were good predictors of post-operative apnea. Sevoflurane anesthesia increased the risk of apnea more than two-fold. The first apnea occurred at a mean of 11 hours post-operatively (range 6-13 hours) and the last apnea occurred at a mean of 24 hours post-operatively (range 6 - 56 hours). These authors recommended overnight observation in selected high-risk infants. It was predicted that even if half of the infants are treated outside NICU, the cost saved annually could exceed $100,000. 

In the act of balancing competing and contradicting needs, Athena would favour safety over cost saving. She is glad that all the recent papers agree towards overnight observation; but they differ in defining criteria of overnight admission. Athena would adopt a liberal policy of overnight hospitalization in all infants undergoing herniotomies. She remembers the old saying that there are no minor surgeries; but there are minor surgeons!

**Contralateral Exploration**

The controversy of contralateral exploration in unilateral hernia is an unending debate. A faction of pediatric surgeons believe that routine contralateral exploration is beneficial as it avoids another operation should metachronous hernia (MH) occurs at a later date. Recently, this notion has patronage from laparoscopic enthusiasts. In opposition, others believe that patent sac need not always cause MH. According to them, routine contralateral exploration is a form of overtreatment that has invisible perils such as potential injury to both side vas, prolonged operating time, increased post-operative apnea, unwarranted increase in the cost of healthcare and needless tissue invasion. 

In this background of discordant opinions, Marulaiah et al [16] conducted a prospective study to deduce the need of contralateral inguinal exploration in premature neonates. About 6% of term neonates and 10% premature newborn had MH. This difference was statistically not significant. Side of initial presentation, sex of newborn, age at surgery and presentation with incarceration had no predictive value for MH. They, therefore, concluded that routine contralateral exploration is not justified in unilateral inguinal hernia of preterm infants. A similar conclusion was independently reached by Steven et al. [17]

**Endnote**

Finally, Athena has occasionally seen ipsilateral hydrocele following herniotomy in neonates and infants. She used to wonder if it could be considered as recurrence or treatment failure. Davies et al [18] reported 5 such neonates. Two of them subsided spontaneously and one resolved with needle aspiration of the fluid. The remaining two underwent surgical exploration; but recurrent or residual hernial sac could not be found in both of them. Athena learns that “watchful expectancy and masterly inactivity” is suitable for hydrocele complicating neonatal herniotomy. 

## Footnotes

**Source of Support:** Nil

**Conflict of Interest:** The author is Editor of the journal. But he did not take part in the evaluation or decision making of this manuscript. The manuscript has been independently handled by two other editors.

